# Supplementary material for: New Anti-inflammatory Flavonol Glycosides from Lindera akoensis Hayata
Source: Molecules. 2019 Feb 4;24(3):563. doi: 10.3390/molecules24030563 (PMC6384965; doi:10.3390/molecules24030563)
Supplement: Supplementary file 1 [file molecules-24-00563-s001.pdf]

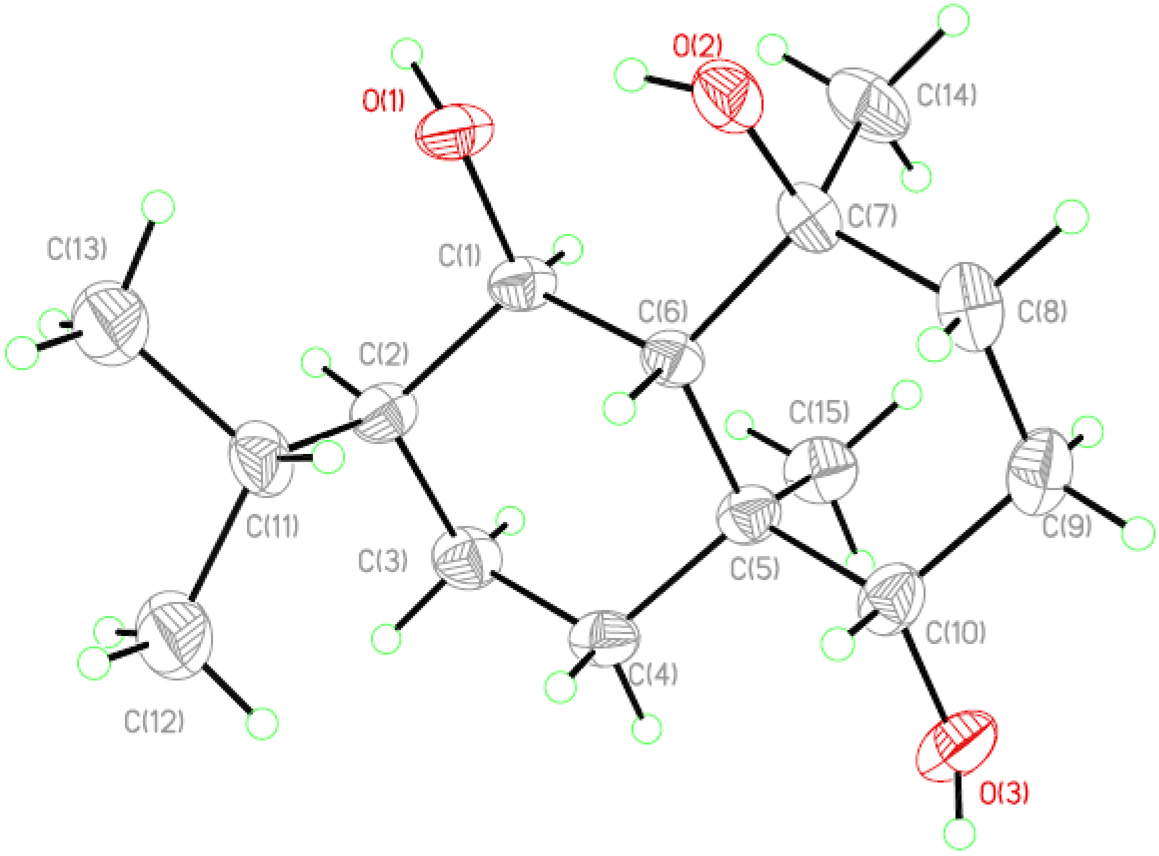

Figure S1. X-ray crystallographic structure of compound **6**.

Table S1. ^{1}H-NMR spectroscopic data of compounds **4–6** (in $CDCl_3$, 400 MHz)[a]

position	**4**	**5**	**6**
1	-	-	3.32 (1H, *m*)
2	-	4.83 (1H, *dd*, *J*= 8.7, 5.9)	1.70 (1H, *m*)
3	2.55 (1H, *m*)	1.77 (1H, *dd*, *J*= 12.4, 5.9)<br>1.52 (1H, *m*)	1.60 (1H, *m*)<br>1.72 (1H, *m*)
4	4.30 (1H, *dd*, *J*= 4.6, 3.0)	-	-
5	4.44 (1H, *dq*, *J*= 3.0, 6.4)	1.45 (1H, *m*)	1.72 (1H, *d*, *J*= 11.2)
6	1.41 (3H, *d*, *J*= 6.4)	1.35 (1H, *m*)<br>1.45 (1H, *m*)	4.33 (1H, *dd*, *J*= 11.2, 4.4)
7	1.23 (2H, *m*)	1.11 (1H, *m*)<br>1.35 (1H, *m*)	2.03 (1H, *m*)
8	1.23 (2H, *m*)	-	1.30 (1H, *m*)<br>1.68 (1H, *m*)
9	1.23 (2H, *m*)	3.29 (1H, *br s*)	1.28 (1H, *m*)<br>1.50 (1H, *m*)
10	1.23 (2H, *m*)	1.15 (1H, *m*)<br>1.58 (1H, *m*)	-
11	1.23 (2H, *m*)	1.55 (1H, *m*)<br>1.95 (1H, *m*)	1.08 (1H, *m*)
12	1.23 (2H, *m*)	1.00 (1H, *m*)<br>1.50 (1H, *m*)	1.09 (3H, *d*, *J*= 6.6)
13	1.23 (2H, *m*)	1.03 (3H, *s*)	0.92 (3H, *d*, *J*= 6.6)
14	1.23 (2H, *m*)	0.89 (3H, *s*)	0.94 (3H, *s*)
15	1.23 (2H, *m*)	0.93 (3H, *s*)	1.34 (3H, *s*)
16	1.23 (2H, *m*)	-	-
17	1.23 (2H, *m*)	-	-
18	0.87 (3H, *t*, *J*= 7.2)	-	-
2′		2.02 (3H, *s*)	

[a]The chemical shifts are expressed in δ ppm. The coupling constants (*J*) are expressed in Hz.

Table S2. ^{13}C-NMR spectroscopic data of compounds **5–6** (in $CDCl_3$, 100 MHz)

position	**5**	**6**
1	44.3	79.4
2	82.1	28.1
3	44.3	40.1
4	38.0	72.6
5	50.3	50.2
6	20.8	73.7
7	33.1	47.4
8	34.6	22.8
9	74.9	35.6
10	27.3	40.7
11	26.3	25.2
12	35.4	22.8
13	31.4	22.3
14	25.3	14.0
15	28.2	24.4
1′	171.0	-
2′	21.3	-
